# Cbl-b inhibits P-gp transporter function by preventing its translocation into caveolae in multiple drug-resistant gastric and breast cancers

**DOI:** 10.18632/oncotarget.3253

**Published:** 2015-02-17

**Authors:** Ye Zhang, Xiujuan Qu, Yuee Teng, Zhi Li, Ling Xu, Jing Liu, Yanju Ma, Yibo Fan, Ce Li, Shizhou Liu, Zhenning Wang, Xuejun Hu, Jingdong Zhang, Yunpeng Liu

**Affiliations:** ^1^ Department of Medical Oncology, the First Hospital of China Medical University, Shenyang 110001, China; ^2^ Department of Surgical Oncology and General Surgery, the First Hospital of China Medical University, Shenyang 110001, China; ^3^ Department of Medical Respiratory, the First Hospital of China Medical University, Shenyang 110001, China

**Keywords:** Cbl-b, P-gp, Caveolae, Multiple Drug-resistant

## Abstract

The transport function of P-glycoprotein (P-gp) requires its efficient localization to caveolae, a subset of lipid rafts, and disruption of caveolae suppresses P-gp transport function. However, the regulatory molecules involved in the translocation of P-gp into caveolae remain unknown. In the present study, we showed that c-Src dependent Caveolin-1 phosphorylation promoted the translocation of P-gp into caveolae, resulting in multidrug resistance in adriamycin resistant gastric cancer SGC7901/Adr and breast cancer MCF-7/Adr cells. In a negative feedback loop, the translocation of Cbl-b from the nucleus to the cytoplasm prevented the localization of P-gp to caveolae resulting in the reversal of MDR through the ubiquitination and degradation of c-Src. Clinical data showed a significant positive relationship between Cbl-b expression and survival in P-gp positive breast cancer patients who received anthracycline-based chemotherapy. Our findings identified a new regulatory mechanism of P-gp transport function in multiple drug-resistant gastric and breast cancers.

## INTRODUCTION

P-glycoprotein (P-gp), which is encoded by the multidrug resistance (MDR) gene 1, is a transmembrane protein that is ubiquitously expressed in normal cells such as the blood brain barrier, liver, bile ducts and kidney cells [1]. P-gp is an ATP-dependent efflux pump that secretes metabolites and removes toxins and drugs to protect normal tissues from damage and maintain homeostasis. However, the protective effect of P-gp is also an important reason for chemotherapy failure. P-gp is overexpressed in tumor tissues in approximately 30% of cancer patients [2], and its expression is induced by repeated exposure to chemotherapeutic drugs. P-gp functions by expelling chemotherapeutic drugs such as anthracyclines, alkaloids and taxanes from the tumor cells, which is the basis of MDR, a phenomenon by which tumor cells, upon contact with one anticancer agent, become resistant to other unrelated agents. Although several agents have been shown to effectively reverse P-gp-mediated MDR, none of these has been successfully developed for clinical use.

The function of P-gp as an efflux pump requires efficient channel formation in the plasma membrane [1, 3]. Lipid rafts are specialized regions of the plasma membrane that are essential for the formation of P-gp channels and play an important role in the modulation of P-gp transport activity [4–6]. Caveolae, a type of lipid raft, are flask-like invaginations of the plasma membrane that serve as organizing centers for cellular signal transduction [7]. Caveolin-1 (Cav-1) is the main protein constituent of caveolae, and *in vitro* studies have shown that P-gp interacts with Cav-1 through a caveolin-binding motif at amino acids 36–44 of P-gp [8, 9]. In an *in vitro* model of the blood-brain barrier, > 50% P-gp was shown to localize to caveolae, and disruption of caveolae led to the suppression of P-gp transport activities [10]. In drug resistant cancer cells, P-gp was shown to be upregulated and localized to caveolae in association with its function in mediating MDR [11]. Therefore, caveolae provide a critical platform for P-gp, and the translocation of P-gp into caveolae might be a regulatory switch for its transport activity. Despite the importance of caveolae in membrane transport, studies on the translocation of functional molecules into caveolae are limited. Bizet et al. reported that CD109 increases binding of TGF-β to its receptor and enhances its translocation into caveolae [12]. Dittmann et al. reported that Src kinase activation following irradiation triggers Cav-1 dependent EGFR internalization into caveolae. However, little is known about the molecules regulating the translocation of P-gp into caveolae [13].

The ubiquitin-proteasome system (UPS) is the main intracellular pathway modulating protein turnover. The UPS regulates the activity of P-gp by modulating P-gp levels [14]. Katayama et al. reported that the E3 ubiquitin ligase FBXO15 ubiquitinates P-gp and targets it for degradation [15]. Recent work from our group demonstrated that Cbl-b, a member of the Cbl family of E3 ubiquitin ligases, downregulates P-gp expression by suppressing Akt activation [16], suggesting that the UPS regulates P-gp levels and thus its function. However, other studies showed that the UPS modulates P-gp function by regulating its localization to lipid rafts. The E3 ligase Trc8 inhibits the transporter function of P-gp by inhibiting the localization of Pgp and MRP1 to lipid rafts and cholesterol synthesis [17]. We previously reported that Cbl-b is also a strong inhibitor of lipid raft aggregation, and prevents EGFR and its related signaling molecules from translocating into caveolae [18, 19]. Therefore, we speculated that Cbl-b might play a role in regulating the transport activity of P-gp by modulating its translocation into caveolae.

In the present study, we showed that c-Src dependent Cav-1 phosphorylation promoted the translocation of P-gp into caveolae, inducing MDR in gastric cancer SGC7901/Adr and breast cancer MCF-7/Adr cells. The translocation of Cbl-b from the nucleus to the cytoplasm prevented the translocation of P-gp into caveolae and reversed MDR through the ubiquitination and degradation of c-Src. Analysis of clinical data showed that Cbl-b expression is significantly associated with survival in P-gp positive patients who received anthracycline based chemotherapy. Our findings suggest a novel mechanism regulating the transport function of P-gp.

## RESULTS

### P-gp transport function is dependent on its translocation into caveolae

**Figure 1:**
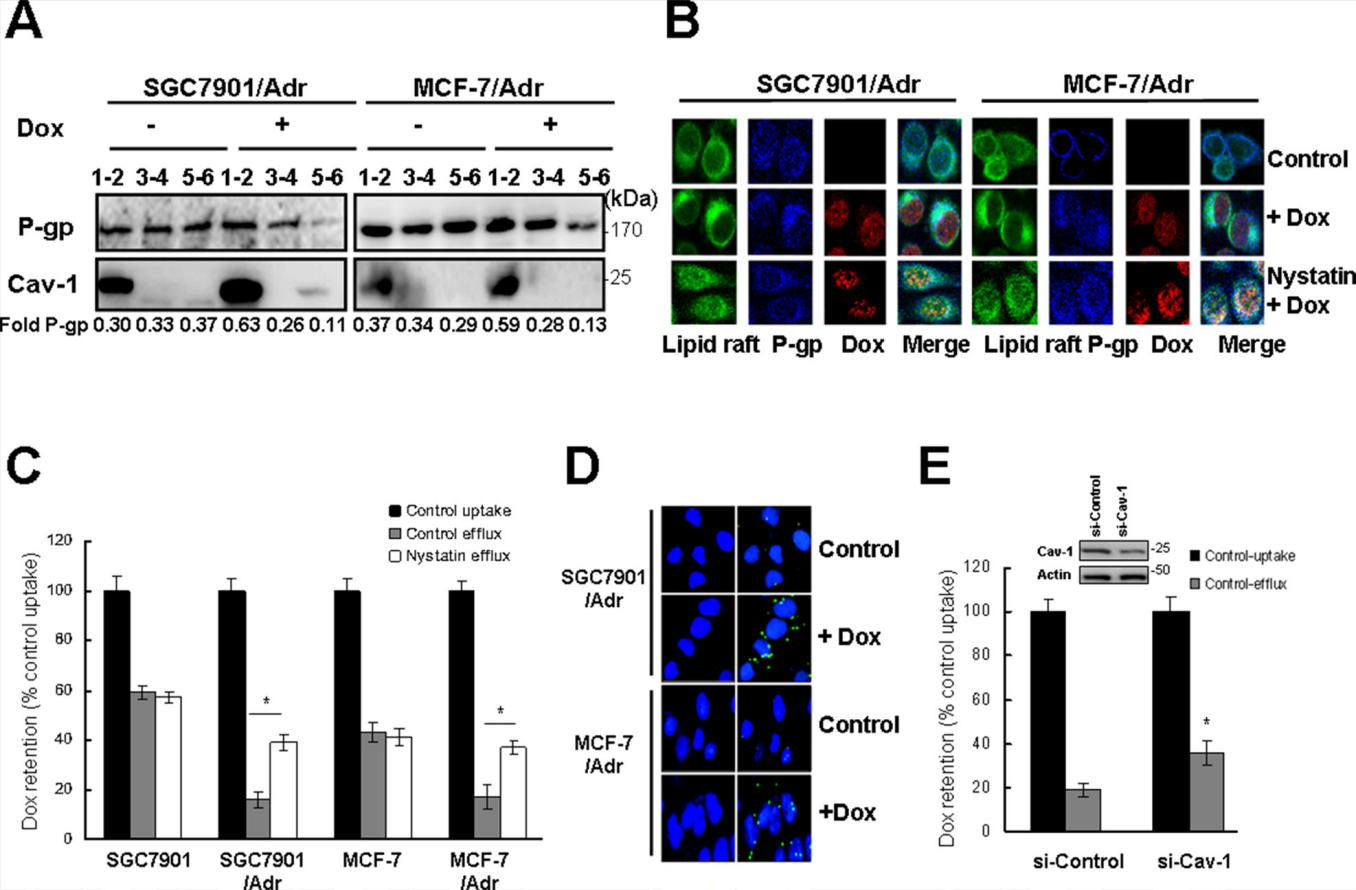
P-gp transport function is dependent on its translocation into caveolae **(A)** SGC7901, SGC7901/Adr, MCF-7 and MCF-7/Adr cells were treated with or without 20 μg/ml Dox for 5 min, lysed and fractionated by ultracentrifugation. P-gp was detected by western blotting. **(B)** Cells were incubated with 50 μg/ml nystatin for 2 h, then treated with 20 μg/ml Dox for 5 min, stained with anti-cholera toxin B subunit or anti-P-gp antibody, and analyzed by confocal fluorescence microscopy. Original magnification, × 40. **(C)** Cells were treated with nystatin (50 μg/ml) and R-123 uptake and efflux were examined in cells treated as in (A). **(D)** *In situ* PLA in SGC7901/Adr and MCF-7/Adr cells treated with or without 20 μg/ml Dox for 5 min. Primary antibodies against P-gp and Cav-1 were combined with secondary PLA probes. **(E)** SGC7901/Adr cells were transiently transfected with Cav-1 siRNA (si-Cav-1) for 48 h, followed by 20 μg/ml Dox and measurement of Dox uptake and efflux.

To determine the role of lipid rafts in P-gp-mediated MDR, lipid raft and non-lipid raft components were separated by sucrose density gradient centrifugation. The results showed that 20 μg/ml Dox increased the levels of P-gp in lipid rafts from 30% to 63% in SGC7901/Adr gastric cancer cells (Figure 1A). Similar results were obtained in MCF-7/Adr breast cancer cells (Figure 1A). Immunofluorescence analysis confirmed the Dox induced translocation of P-gp to aggregated lipid rafts, and this was inhibited by treatment with 50 μg/ml nystatin, a cholesterol-sequestering agent that disrupts lipid rafts (Figure 1B). P-gp function was assessed by measuring Dox uptake and retention, which showed that pretreatment with 50 μg/ml nystatin increased retention and inhibited the release of Dox and sensitized SGC7901/Adr and MCF7/Adr cells to Dox (Figure 1C). These findings suggest that P-gp transport function is dependent on its localization to lipid rafts.

Because caveolae are a type of lipid raft that play an important role in signal transduction, we investigated their potential as a target domain for the translocation of P-gp by detecting the interaction between P-gp and Cav-1, a marker of caveolae, using the PLA. The results showed that treatment with 20 μg/ml Dox for 5 min promoted a significant and rapid interaction between P-gp and Cav-1 in SGC7901/Adr and MCF-7/Adr cells (Figure 1D). Small interfering RNA (siRNA) mediated knock-down of Cav-1 markedly increased the intracellular retention of Dox in SGC7901/Adr cells (Figure 1E). These data suggested that the translocation of P-gp into caveolae is necessary for MDR in cancer cells.

### c-Src dependent Cav-1 phosphorylation promotes the translocation of P-gp into caveolae

**Figure 2:**
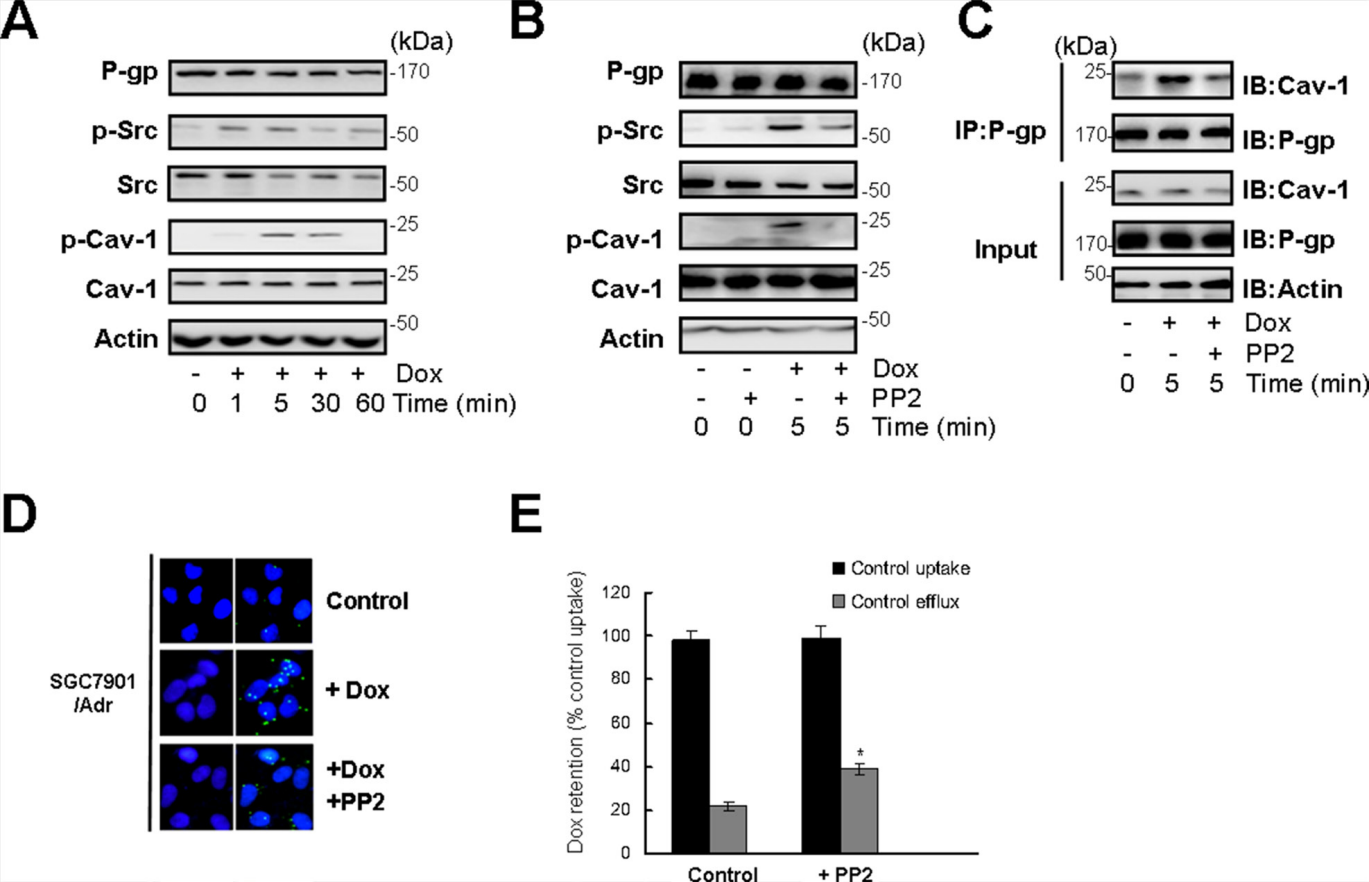
c-Src dependent Cav-1 phosphorylation promoted the translocation of P-gp into caveolae **(A)** SGC7901/Adr cells were treated with or without 20 μg/ml Dox for 1, 5, 30, 60 min and the expression of P-gp, p-Src, Src, p-Cav-1, Cav-1 and Actin was detected by western blotting. **(B)** Cells were incubated with the Src inhibitor PP2 (10 μmol/l) for 2 h, treated with 20 μg/ml Dox for 5 min, and the expression of P-gp, p-Src, Src, p-Cav-1, Cav-1 and Actin was detected by western blotting. **(C)** SGC7901/Adr cells were pretreated with 10 μmol/l PP2 for 2 h followed by Dox treatment, and P-gp was immunoprecipitated and Cav-1 was analyzed western blotting. **(D)** *In situ* PLA in SGC7901/Adr cells pretreated with or without 10 μmol/l PP2 for 2 h, and then incubated with 20 μg/ml Dox for 5 min. Primary mouse and rabbit antibodies against P-gp and Cav-1 were combined with secondary PLA probes. **(E)** SGC7901/Adr cells were pretreated with 10 μmol/l PP2 for 2 h, followed by 20 μg/ml Dox and assessment of R-123 uptake and efflux.

Because the Src family of tyrosine kinases is present in lipid rafts, we detected c-Src activation in response to Dox treatment in SGC7901/Adr cells. The results indicated that 20 μg/ml Dox triggered a transient activation of c-Src, which reached a peak at 5 min despite a decrease in total c-Src levels (Figure 2A). Cav-1 was activated at 5 min, which was later than c-Src activation. PP2, a specific inhibitor of c-Src kinase, markedly suppressed Cav-1 phosphorylation (Figure 2B). Immunoprecipitation of P-gp and detection of Cav-1 showed that Dox treatment promoted P-gp/Cav-1 complex formation after 5 min, and PP2 suppressed the formation of the P-gp/Cav-1 complex (Figure 2C). Immunofluorescence assays further confirmed that PP2 prevented the interaction between P-gp and Cav-1 (Figure 2D). Consequently, the intracellular retention of Dox was increased in SGC7901/Adr cells (Figure 2E). These results indicated that c-Src promoted the translocation of P-gp into caveolae by activating Cav-1 and promoted the retention of Dox in SGC7901/Adr and MCF-7/Adr cells.

### Cbl-b inhibited the translocation of P-gp into caveolae through the ubiquitination and degradation of c-Src

**Figure 3:**
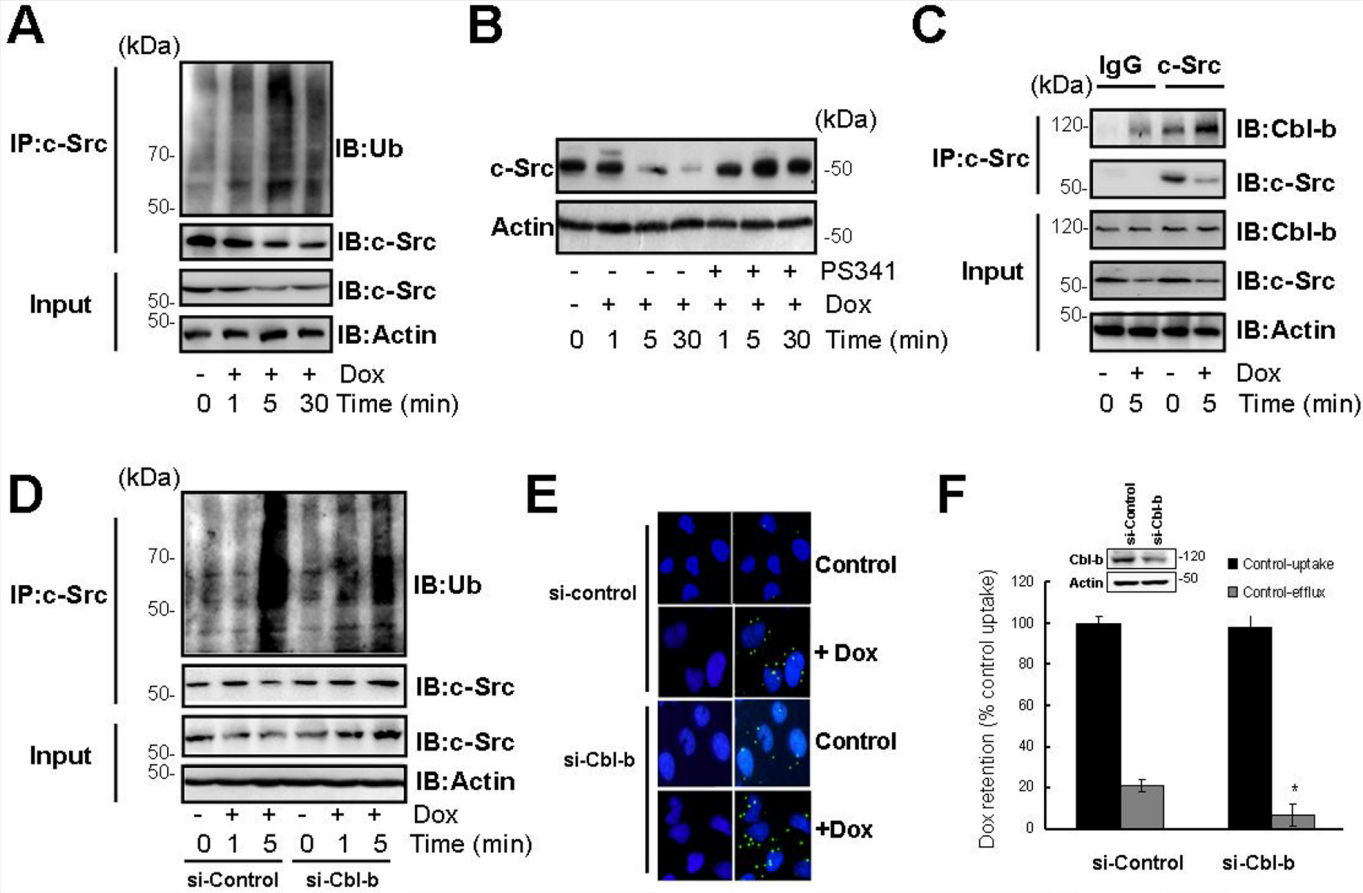
Cbl-b inhibited the translocation of P-gp into caveolae by inducing the ubiquitination and degradation of c-Src **(A)** SGC7901/Adr cells were exposed to 20 μg/ml Dox for 1, 5, 30 min, c-Src was immunoprecipitated and ubiquitin was analyzed by western blotting. **(B)** SGC7901/Adr cells were incubated with PS341 (5 nmol/l) for 12 h, then treated with 20 μg/ml Dox for 5 min, and the expression of the Src protein was analyzed by western blotting. **(C)** SGC7901/Adr cells were treated with or without 20 μg/ml Dox for 5, c-Src was immunoprecipitated and Cbl-b was analyzed by western blotting. **(D)** SGC7901/Adr cells were transiently transfected with Cbl-b siRNA (si-Cbl-b) for 48 h, followed by 20 μg/ml Dox for 1 or 5 min, c-Src was immunoprecipitated and ubiquitin was analyzed by western blotting. **(E)** SGC7901/Adr cells were transiently transfected with Cbl-b siRNA (si-Cbl-b) for 48 h and analyzed by PLA after incubation with 20 μg/ml Dox for 5 min. Primary mouse and rabbit antibodies against the P-gp and Cav-1 were combined with secondary PLA probes. **(F)** SGC7901/Adr cells were transiently transfected with Cbl-b siRNA (si-Cbl-b) for 48 h, followed by 20 μg/ml Dox and analysis of Dox uptake and efflux.

Because ubiquitination serves as a signal for protein degradation, ubiquitination of c-Src was tested after exposure of SGC7901/Adr cells to 20 μg/ml Dox for the indicated times. Polyubiquitinated c-Src was detected within 5 min (Figure 3A). Pretreatment with the proteasome inhibitor PS34 effectively prevented c-Src degradation in SGC7901/Adr cells (Figure 3B). These findings suggested that Dox promoted the degradation of c-Src in a UPS dependent manner.

Cbl-b is an E3 ubiquitin ligase that binds to numerous proteins and mediates their ubiquitination and degradation. To determine whether Cbl-b is involved in the regulation of c-Src stability, SGC7901/Adr cells were treated with 20 μg/ml Dox for the indicated times and the interaction between c-Src and Cbl-b was demonstrated by co-immunoprecipitation (Figure 3C).

To determine whether c-Src is ubiquitinated by Cbl-b in response to Dox, Cbl-b was silenced in SGC7901/Adr cells by transfection with siRNA Cbl-b, which significantly inhibited Dox-induced ubiquitination and degradation of c-Src (Figure 3D). This result was confirmed by overexpression of wild-type Cbl-b, which significantly enhanced Dox-induced c-Src ubiquitination. Cbl-b (C373A), which lost its ubiquitin-protein ligase function by a point mutation of Cys373 to Ala, abolished this enhancement (Supplementary Figure 2A–2B, available online). Protein interactions between P-gp and Cav-1 were tested by the PLA. The results showed that knockdown of Cbl-b significantly increased the interaction between P-gp and Cav-1 in SGC7901/Adr cells (Figure 3E), and promoted the release of Dox (Figure 3F). These results indicated that Cbl-b could inhibit the transport activity of P-gp through the ubiquitination and degradation of c-Src.

**Figure 4:**
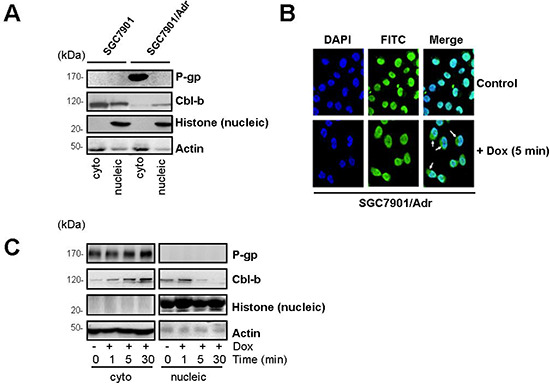
Dox induced the translocation of Cbl-b from the nucleus to the cytoplasm **(A)** Western blot analysis of cytoplasmic and nuclear fractions from SGC7901 and SGC7901/Adr cells. Nuclear fractionation was confirmed by detection of total histone. Cytoplasmic fractionation was confirmed by detection of actin. **(B)** Immunofluorescence microscopy analysis of cytoplasmic and nuclear Cbl-b expression. **(C)** Western blot analysis of cytoplasmic and nuclear fractions from SGC7901/Adr cells treated with 20 μg/ml Dox for 1, 5, and 30 min. Nuclear fractionation was confirmed by detection of total histone and cytoplasmic fractionation by actin.

The localization of Cbl-b in P-gp positive and negative cells was analyzed by western blotting (Figure 4A). In SGC7901/Adr cells, Cbl-b was mainly detected in the nucleus, whereas in parental SGC7901 cells, it was mostly detected in the cytoplasm (Figure 4B). Western blotting also showed that Dox (20 μg/ml) rapidly triggered Cbl-b translocation from the nucleus to cytoplasm in a time-dependent manner (Figure 4C). Low dose Dox (2 μg/ml) could not trigger Cbl-b translocation from the nucleus to the cytoplasm (Supplementary Figure 3, available online).

### Cbl-b increased Dox therapeutic sensitivity in xenografts and prolonged remission and disease-specific survival in P-gp positive breast cancer patients treated with an anthracycline-based regimen

**Figure 5:**
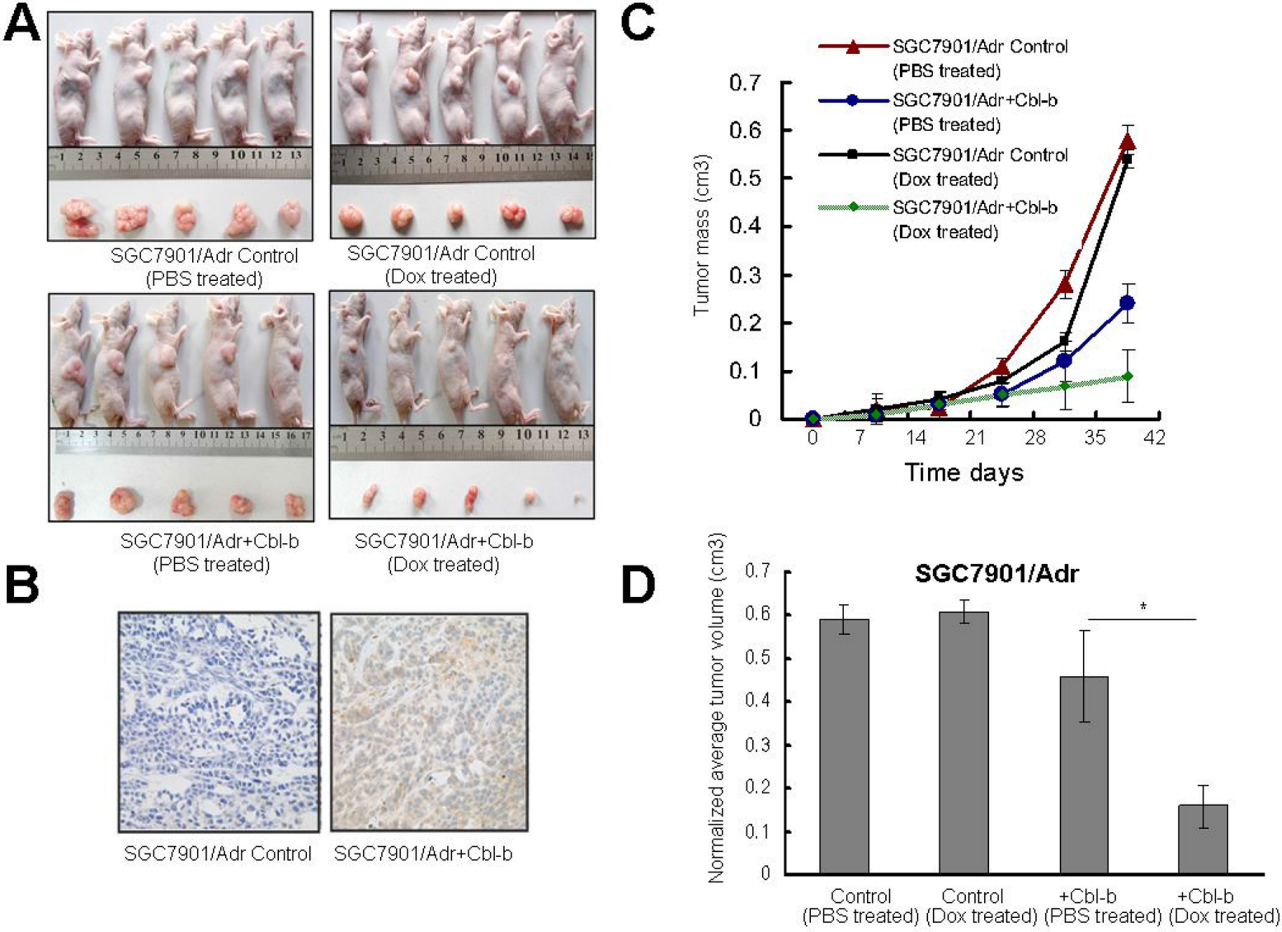
Effect of Cbl-b on Dox therapeutic sensitivity of subcutaneous gastric cancer in mice **(A)** Effect of Cbl-b overexpression on tumor proliferation. SGC7901/Adr Control and SGC7901/Adr+Cbl-b were implanted subcutaneously into the right flanks of 4–6-week-old female nude mice (*n* = 5 mice per group), and tumor volume was measured on days 0, 8, 16, 24, 32 and 40 after implantation. **(B)** Cbl-b expression was analyzed in sections from subcutaneous SGC7901/Adr control and SGC7901/Adr+Cbl-b tumors by immunohistochemistry (× 20). **(C–D)** Average normalized tumor volume from mice (*n* = 5 per group) at the experimental end-point. Mean volumes and error bars representing 95% confidence intervals from three independent experiments are shown. *P = 0.34, SGC7901/Adr+Cbl-b treated with Dox vs. SGC7901/Adr+Cbl-b treated with PBS; *P = 0.12, SGC7901/Adr+Cbl-b treated with Dox vs. SGC7901/Adr Control treated with PBS; *P = 0.09, SGC7901/Adr+Cbl-b treated with Dox vs. SGC7901/Adr Control treated with Dox.

The findings that Cbl-b could overcome Dox resistance *in vitro* were further explored by *in vivo* nude mice xenograft studies. Stable SGC7901/Adr cell lines overexpressing Cbl-b/pcDNA3.1 or pcDNA3.1 alone were subcutaneously implanted into the flanks of athymic nude mice. After tumors reached approximately 100 mm^3^ (7 days), the animals received intraperitoneal injection of either PBS or Dox (6 mg/kg) once per week. The results showed that in SGC7901/Adr+Cbl-b mice treated with Dox, tumor growth was significantly inhibited compared with that in the other three groups (Figure 5A–5D).

**Figure 6:**
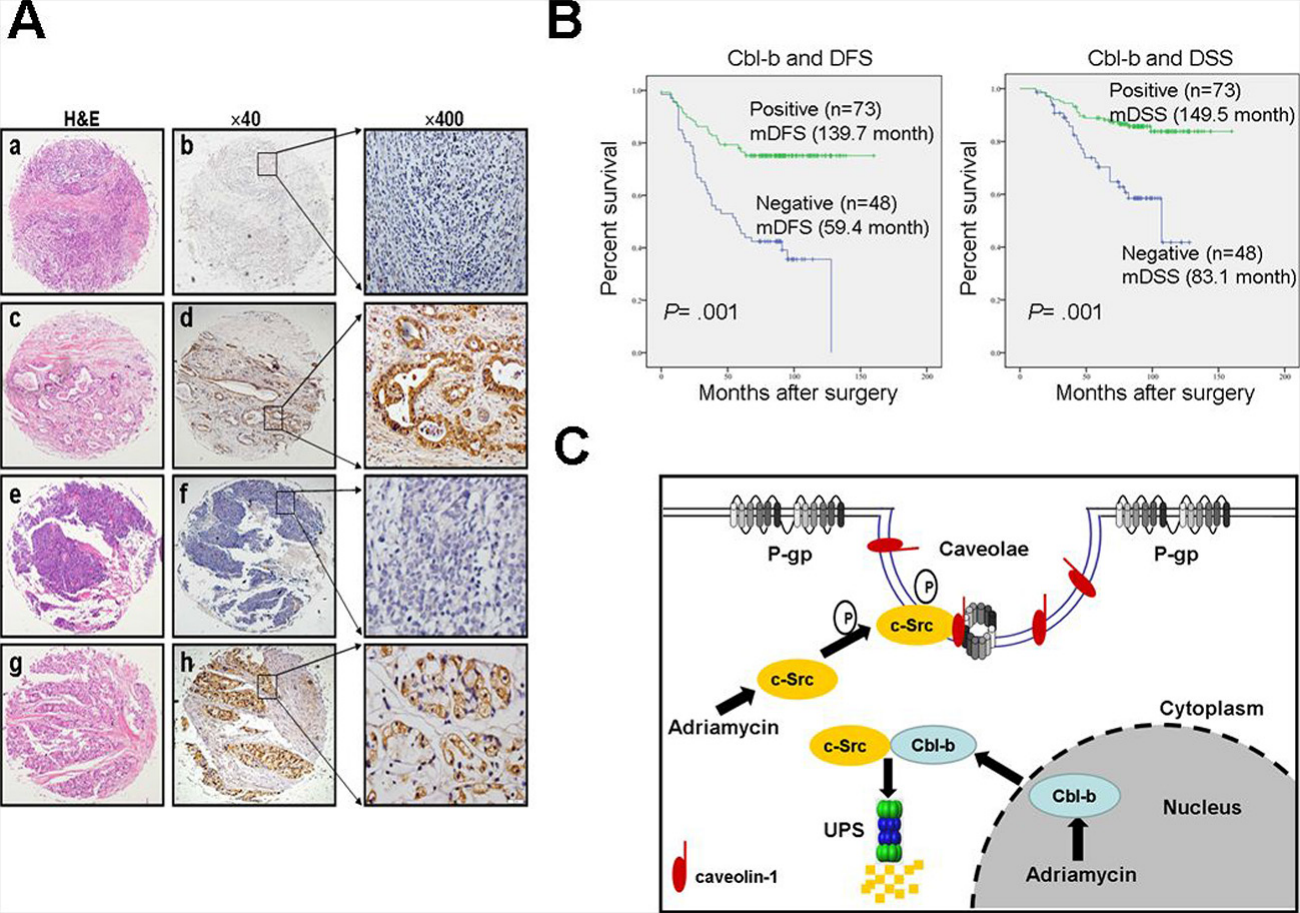
Cbl-b prolonged remission and disease-specific survival in P-gp positive breast cancer patients treated with a Dox-based regimen **(A)** Immunohistochemistry images of tissue sections stained against Cbl-b and P-gp. a–d shows breast tissue negatively and positively stained with anti-Cbl-b antibody; **e–h** show breast tissue negatively and positively stained with anti-P-gp antibody. **(B)** Disease-specific survival rates of patients with P-gp positive breast cancer positive or negative for Cbl-b were estimated with the Kaplan–Meier method and log-rank test; *n* = 121, *P* < 0.05. **(C)** Proposed model showing that c-Src dependent Cav-1 phosphorylation promoted the translocation of P-gp into caveolae, resulting in MDR. In a negative feedback loop, Cbl-b translocated from the nucleus to the cytoplasm, preventing the translocation of P-gp into caveolae and reversed MDR by ubiquitinating c-Src and targeting it for degradation.

We also determined whether Cbl-b expression was associated with the clinical outcomes of patients with breast cancer. To this end, tumor samples from 211 breast cancer patients were collected for immunohistochemistry using anti-Cbl-b and anti-P-gp antibodies (Figure 6A). The levels of P-gp had no significant influence on survival in cancer patients undergoing chemotherapy (Supplementary Figure 4, available online). Survival analysis was performed in 121 patient samples (37.9%) that were positive for P-gp, which showed a statistically significant difference in disease-free survival (DFS) and disease-specific survival (DSS) between 73 patients whose tumors were positive for Cbl-b expression and 48 patients whose tumors were negative for Cbl-b expression as estimated with the Kaplan–Meier method and log-rank test (Table 1, Figure 6B). Our results indicate that decreased expression of Cbl-b is associated with poor prognosis in patients with drug resistant breast cancer. Taken together, these findings suggest that Cbl-b may play a role in tumor MDR.

**Table 1:** Relationship between the expression of Cbl-b and clinico-pathological characteristics of P-gp-positive gastric cancer patients

Clinico-pathological characteristics		Number	Cbl-b expression		*p* value
			Negative	Positive	
Age(years)					
	≤ 35	7	5	2	
	> 35	114	43	71	0.112
Tumour size (cm)					
	≤ 2	19	9	10	
	> 2	102	39	63	0.455
pN stage					
	0	58	19	39	
	1–3	63	29	34	0.136
Histology grade					
	1 + 2	87	33	54	
	3	34	15	19	0.532
ER/PR status^§^					
	Negative	59	19	40	
	Positive	62	29	33	0.101
HER2 status^¶^					
	Negative	39	22	17	
	Positive	82	26	56	**0.009^1^**

1 Two-sided *p* value; values shown in bold are statistically significant.

§ ER/PR status: oestrogen receptor or progesterone receptor status by immunohistochemistry (IHC); negative: ER and PR double negative; positive: ER or PR positive.

¶ HER2 status: HER2-positive status is IHC 3+ or fluorescence *in situ* hybridisation (FISH) positive; HER2-negative status is IHC 0, 1+ or FISH negative.

HER2, Human epidermal growth factor receptor 2.

## DISCUSSION

The localization of P-gp to caveolae is necessary for its transport function; however, the molecules regulating its localization have not been identified. The localization of receptor proteins such as VEGFR2 and EGFR to caveolae depends on their interaction with Cav-1, and phosphorylation of Cav-1 is required for this interaction [20, 21]. To date, c-Src is the only kinase known to phosphorylate Cav-1 [22]. c-Src phosphorylates Cav-1 and promotes its interaction with VEGFR2 or EGFR, leading to their localization to caveolae [23, 24]. In the present study, we showed that treatment with Dox triggered the phosphorylation of Cav-1 by c-Src and the translocation of P-gp to caveolae. PP2, a specific inhibitor of c-Src, inhibited Cav-1 phosphorylation and prevented the translocation of P-gp, simultaneously increasing intracellular retention of Dox in the adriamycin resistant cell lines SGC7901/Adr and MCF-7/Adr. These findings suggested that c-Src-dependent Cav-1 phosphorylation drives the translocation of P-gp into caveolae, eventually resulting in MDR.

In the present study, we identified a novel feedback regulatory mechanism for the translocation of P-gp into caveolae. c-Src dependent Cav-1 phosphorylation is necessary for the translocation of P-gp into caveolae; therefore, molecules affecting the expression and activation of c-Src could play a regulatory role in this translocation. The stability of c-Src is regulated by the UPS in some cells [25, 26], and the E3 ligase c-Cbl was shown to ubiquitinate and target c-Src for degradation in response to TRAIL treatment [27]. c-Cbl and Cbl-b belong to the same ubiquitin ligase family, and have a similar protein domain structure [28], implying that Cbl-b might also be involved in the regulation of c-Src. In the present study, Dox treatment promoted the rapid downregulation of c-Src in a UPS dependent manner. Knockdown of Cbl-b significantly inhibited c-Src ubiquitination and degradation and promoted the translocation of P-gp to caveolae, whereas Cbl-b overexpression prevented the translocation of P-gp to caveolae. These results indicated that Cbl-b may reverse P-gp-mediated MDR by suppressing the c-Src/Cav-1 induced translocation of P-gp to caveolae.

As a cytosolic protein, c-Src normally functions in the proximity of or in the plasma membrane. In the present study, we showed that a significant proportion of Cbl-b localized to the nucleus in untreated MDR cells. A potential nuclear localization signal in Cbl-b was reported by Keane MM [28]. Since these two proteins localize to different cellular compartments, the mechanism underlying the regulation of c-Src by Cbl-b is not clear. Furthermore, our data showed that Cbl-b translocates from the nucleus to the cytoplasm in response to Dox treatment. Cytoplasmic Cbl-b promoted c-Src ubiquitination and degradation, and inhibited the translocation of P-gp into caveolae, resulting in the reversal of MDR. To date, there are no reports describing the nuclear localization of Cbl-b or that of other Cbl family proteins. The present study is the first to show the nuclear localization and Dox-induced nucleus to cytoplasm translocation of Cbl-b. However, elucidation of the mechanism of Cbl-b translocation requires further investigation.

P-gp overexpression is an important mechanism of MDR and it has been described in numerous studies. However, previous clinical data suggest that the levels of P-gp have no significant influence on survival in cancer patients undergoing chemotherapy [29]. This implies that other as yet unidentified factors may be involved in the regulation of P-gp function and affect the survival of P-gp positive patients. Our *in vitro* and animal experiments confirmed the effect of P-gp overexpression on the development of MDR. However, our clinical data showed that P-gp alone had no effect on DFS or DSS in breast cancer patients who received anthracycline-based chemotherapy, whereas Cbl-b significantly prolonged the survival of P-gp positive patients. Our previous studies showed that Cbl-b reverses P-gp-mediated MDR by downregulating P-gp expression. Furthermore, previous studies have shown that Cbl-b reverses P-gp-mediated MDR by inhibiting the translocation of P-gp into caveolae. These results suggest that even in P-gp positive cancer patients, Cbl-b is associated with a survival benefit. Therefore, detection of Cbl-b and P-gp in combination could be a valuable prognostic marker in cancer patients who received anthracycline-based chemotherapy.

In summary, our study provided evidence that c-Src dependent Cav-1 phosphorylation promotes the translocation of P-gp into caveolae, leading to MDR. We also identified a novel negative feedback loop by which the translocation of Cbl-b from the nucleus to the cytoplasm prevents the c-Src/Cav-1 dependent translocation of P-gp into caveolae, overcoming MDR (Figure 6C). Clinical data confirmed that Cbl-b significantly prolonged survival in P-gp positive patients who received anthracycline based regimens. Our findings identified a new regulatory mechanism of P-gp transport activity.

## PATIENTS, MATERIALS, AND METHODS

### Patients and tissue samples

The files of 211 patients who underwent surgical resection of breast cancer and were treated with Dox-based regimens between December 2001 and February 2011 at the Department of Medical Oncology, the First Hospital of China Medical University, were included in our study. Patient information included age, gender, histopathologic type, performance status, metastases, treatment, date of treatment with Dox, cycles of Dox-based chemotherapy, date of progression, and date of death/last follow-up. All patients were treated with Dox-based chemotherapy for a minimum of four cycles. Signed informed consent was obtained from each patient. This study was approved by the Human Ethics Review Committee of China Medical University; informed consent was obtained from all patients in accordance with the Declaration of Helsinki and its later revision.

### Tissue microarray (TMA) construction

TMAs were already available for use in the current study [16] and consisted of 2 mm^2^ cores of breast cancer tissue, identified by the pathologist, that were removed from representative areas of the tumor collected from breast cancer patients at the time of surgical resection. All TMA blocks were constructed in triplicate.

### Immunohistochemistry

Immunohistochemical staining was performed using a streptavidin-peroxidase procedure [16]. Cbl-b and P-gp expression was investigated using antibodies against Cbl-b (Santa Cruz Biotechnology, Santa Cruz, CA, USA) and P-gp (Santa Cruz Biotechnology). All other reagents were from Sigma. The specificity of all antibodies was confirmed by western blotting. For statistical analysis, immunostaining was considered positive when the tumour mass occupied more than 10% of the cross-sectional core area and when 10% or more of the neoplastic cells were stained.

### Scoring

Protein expression in each core (three per tumor specimen) was assessed using the weighted histoscore method [16]. The weighted histoscore system grades staining intensity as negative (0), weak (1), moderate (2), and strong (3), and the percentages of tumor cells within each category are calculated. The histoscore range is from zero (minimum) to 300 (maximum). Agreement between observers was excellent and all interclass correlation coefficient scores were above 0.84.

### Cell culture

The human gastric adenocarcinoma cell line SGC7901 and the human breast carcinoma cell line MCF-7 were purchased from the Academy of Military Medical Science (Beijing, China). The adriamycin-resistant variant of SGC7901 (SGC7901/Adr) was kindly provided by the Fourth Military Medical University (Xi’an, China). The adriamycin-resistant variant of MCF-7 (MCF-7/Adr) was established by selecting for resistant colonies by first culturing the parent cell line in 0.2 μg/ml adriamycin (Dox). The drug-containing medium was changed weekly, and colonies that had propagated from single cells were selected. Clonal cell lines expanded from such a colony were subsequently selected in 1 μg/ml Dox (MCF-7/Adr).

All cell lines were grown at 37°C and in 5% CO_2_. SGC7901 and SGC7901/Adr cells were maintained in RPMI 1640 (Invitrogen, Grand Island, NY, USA) supplemented with 10% fetal calf serum (Invitrogen), 100 U/ml penicillin, and 100 mg/ml streptomycin. MCF-7 and MCF-7/Adr cells were maintained in MEM media (Invitrogen) supplemented with 10% fetal calf serum, 100 U/ml penicillin, 100 mg/ml streptomycin, 2 mM L-glutamine, 0.1 mM non-essential amino acids, 1 mM sodium pyruvate, and MEM vitamin solution. In the two drug-resistant variant cell lines, the medium additionally contained 1 μg/ml Dox to maintain the drug resistance phenotype (Supplementary Figure 1A–1B, available online).

### Small interfering RNA transfections

Caveolin-1 (Cav-1) small interfering RNA (siRNA) was obtained from Shanghai GeneChem Co. Ltd. (China). Cav-1 siRNA was generated with the target sequence 5′-AACCAGAAGGGACACACAGTT-3′. Lipofectamine 2000 (Invitrogen) was diluted drop-wise into RPMI 1640 and incubated at room temperature for 5 min. Then, Cav-1 siRNA was added to the diluted Lipofectamine 2000 and incubated for 20 min. After 48 h of transient transfection, the cells were analyzed by western blotting against Cav-1 to assess the siRNA effect.

### Construction of DNA

The human Cbl-b cDNA was kindly provided by Dr Stanley Lipkowitz (National Naval Medical Center, Bethesda, MD, USA). The cDNA of Cbl-b was then used to generate its mutant form, which lost its ubiquitin-protein ligase function by a point mutation of Cys373 to Ala (C373A) [19]. All mutations were confirmed by DNA sequencing. All cDNAs were then subcloned into the pcDNA3.1 plasmid (Invitrogen).

### Cell transfection

SGC7901/Adr cells were plated in six-well plates and cultured in drug-free medium. At 90–95% confluence, cells were washed twice with PBS and incubated in 2 ml of RPMI 1640 medium without antibiotics. Transfection of SGC7901/Adr cells was performed using the Lipofectamine 2000 reagent (Invitrogen, Inc., Carlsbad, CA, USA) with 2 μg of pcDNA3.1 plasmid (Invitrogen) including the full-length cDNA for Cbl-b. Cells transfected with the pcDNA3.1 vector alone served as the negative control. Forty-eight hours later, cells were placed in growth medium containing 600 μg/ml of the neomycin analogue G418 (Life Technologies) for clone selection. Expression levels of Cbl-b in stable G418-resistant clones were evaluated by western blotting.

### Isolation of lipid rafts

The cells were solubilised in 150 μL of prechilled TXNE buffer (50 mM Tris–HCl pH 7.4, 150 mM NaCl, 5 mM EDTA and 0.1% Triton X-100) containing protease inhibitors (chymostatin, leupeptin, antipain, and pepstatin, at 25 mg/mL each) for 20 min on ice. Subsequently, the cells were scraped off, extracted and moved into 35% Optiprep (Axis-shield, Norway) in polyallomer ultra tubes (Sorvall Instruments, USA) by adding 210 μL of 60% Optiprep/0.1% Triton X-100. Then the cell lystaes were covered with 3.5 mL 30% Optiprep in TXNE buffer and 300 μL TXNE buffer. After spin (4 h, 20,0000 g, 4°C) in the ultracentrifuge (Sorvall/Kendro, USA), six fractions were collected from the top. The proteins in fractions 1–2 were collected and were taken as the lipid raft fractions.

### Lysate preparation, immunoprecipitation, and immunoblotting

Lysate preparation and protein immunoprecipitation were performed as described previously [18]. Cells were washed twice with ice-cold phosphate-buffered saline (PBS) and solubilized in 1% Triton lysis buffer (1% Triton X-100, 50 mM Tris-HCl [pH 7.4], 150 mM NaCl, 10 mM EDTA, 100 mM NaF, 1 mM Na_3_VO_4_, 1 mM PMSF, 2 μg/ml aprotinin) on ice. For immunoprecipitation with anti–P-gp or anti-c-Src, cells were solubilized in denaturing buffer (1% Triton X-100 lysis buffer containing 0.1% SDS and 0.5% deoxycholic acid) to dissociate protein complexes. Cell lysates were precleared by centrifugation, and the resultant supernatants were incubated with the indicated antibodies prebound to protein A-agarose or protein G-sepharose beads. After rotation for 1 h at 4°C, the beads were washed four times with lysis buffer, and the immunoprecipitated proteins were eluted by heat treatment at 100°C for 5 min with 2 × sampling buffer.

Total cell lysates, immunoprecipitated proteins, and subcellular fractions were separated by SDS-polyacrylamide gel electrophoresis and transferred to polyvinylidene difluoride transfer membranes (Millipore, Bedford, MA, USA). After blocking with 5% skim milk in TBST (10 mM Tris, pH 7.4, 150 mM NaCl, and 0.1% Tween20), the blots were probed with the indicated primary antibodies for 1 h at 4°C. After washing with TBST, the membrane was reacted with the appropriate horseradish peroxidase–conjugated secondary antibodies for 30 min at room temperature. After extensive washing with TBST buffer, the proteins were detected using the enhanced chemiluminescence reagent (SuperSignal Western Pico Chemiluminescent Substrate; Pierce, USA) and visualized with the Electrophoresis Gel Imaging Analysis System (DNR Bio-Imaging Systems, Israel).

### Immunofluorescence microscopy

Cells cultured in Lab-Tek chamber slides (Nunc S/A, Polylabo, Strasbourg, France) were fixed with 3.3% paraformaldehyde/PBS, permeabilized with 0.1% Triton X-100/PBS, and washed with PBS three times. For double staining of lipid rafts and P-gp, the cells were primed with FITC-conjugated rabbit anti-cholera toxin B subunit, anti-P-gp mouse monoclonal antibody for 1 h and then incubated with Alexa Fluor&reg 405 goat anti-mouse IgG for 45 min. Merged images display the overlay of blue P-gp staining of membrane, green lipid rafts staining and red autofluorescence of DOX. Finally, the cells were mounted using the SlowFade Antifade Kit (Molecular Probes, Eugene, OR) and analyzed by confocal fluorescence microscopy (FV1000S-SIM/IX81, Olympus, Japan).

### Dox uptake and retention

The intracellular uptake and retention of Dox in SGC7901/Adr and MCF-7/Adr cells was measured by flow cytometry as previously described [30]. Briefly, cells were seeded at a density of 2 × 10^4^ cells per well in 24-well plates and allowed to attach overnight. After attachment, media were removed and replaced with fresh full-growth media and treatments were added for a 30 min preincubation period. After 30 min, media were replaced with fresh media containing treatments and Dox (20 μg/ml). Cells were then incubated at 37°C for 1 h. For uptake studies, cells were placed on ice at the end of the 1 h Dox uptake incubation period, washed twice with ice-cold Hanks buffer, and intracellular Dox was extracted with 0.1% SDS in PBS. Fluorescence was measured at k excitation 488 nm/k emission 575 nm using a fluorescent microplate reader. For retention assays, following the 1 h Dox uptake incubation, media were removed, cells were washed twice with fresh media, and then fresh media with the respective treatments were added and cells were incubated for a further 2 h. One set of wells without treatment was immediately washed and Dox was extracted following the 1 h uptake incubation to serve as uptake controls. At the end of the 2 h efflux incubation period, intracellular Dox was determined as outlined above. For comparison, the intracellular Dox at the end of the efflux period was compared with the intracellular Dox at the end of the uptake period (100% uptake) for each individual cell line.

### Detection of protein interactions by *in situ* proximity ligation assay (PLA)

Primary antibodies used were mouse anti-P-gp monoclonal antibody (Santa Cruz, CA, USA) and rabbit anti-Cav-1 polyclonal antibody (Santa Cruz, CA, USA). Cells were harvested and pretreated according to standard immunofluoresence staining protocols with modifications, and the PLA was performed as described previously by Soderberg et al. [31]. PLA signals were detected with a Duolink PLA (Green) detection kit.

### Preparation of cytosolic and nuclear proteins

The cytosolic and nuclear proteins were prepared as described previously [32]. Briefly, the cell pellets (1 × 10^6^ cells) were treated with 100 μl of hypotonic buffer (10 mM HEPES at pH 7.8, 10 mM KCl, 0.1 mM EDTA, 1 mM dithiothreitol, 0.5 mM phenylmethylsulfonylfluoride [PMSF], 2 μg/ml pepstatin, and 2 μg/ml leupeptin). After centrifugation of the sample (1800 g, 4°C, 1 min), the supernatant and debris were collected as rough cytosolic and nuclear fractions, respectively. The rough cytosolic fractions were centrifuged at 15,000 g for 20 min and 100,000 g for 30 min at 4°C. The final supernatant was collected as the cytosolic fraction for use in the following study. The rough nuclear fractions were washed three times with hypotonic buffer and treated with 100 μl of 50 mM HEPES (pH 7.8), 420 mM KCl, 0.1 mM EDTA, 1 mM dithiothreitol, 5 mM MgCl_2_, 0.5 mM PMSF, 2 μg/ml pepstatin, and 2 μg/ml leupeptin and were then gently rotated with a rotator at 4°C for 30 min. The supernatant was collected as the nuclear fraction.

### Antitumor assays using mouse models

All *in vivo* experiments were approved by the Institutional Review Board of China Medical University. Athymic nude (nu/nu) mice, 4–6 weeks of age, were purchased from SLAC Animal Center (Shanghai, China). SGC7901/Adr-Vector and SGC7901/Adr-Cbl-b cells (1 × 10^6^ in 200 μl PBS) were injected subcutaneously near the scapula of the nude mice. One week after the cells were injected, the mice were randomly separated into two groups, each containing five mice, and treated with PBS or Dox (6 mg/kg) by intraperitoneal injection once per week. Tumors were measured with a caliper every 2 days, and tumor volume was calculated using the formula V = 1/2 (width^2^ × length). Body weights were also recorded. Mice were killed by cervical dislocation when the tumor diameters reached 1.5 cm, according to the protocol filed with the Guidance of Institutional Animal Care and Use Committee of China Medical University.

### Statistical analysis

Data are presented as mean ± standard deviation. The significance of the difference between the groups was assessed by Student's two-tailed *t*-test. A *p* value < 0.05 was considered significant. All means were calculated from at least three independent experiments.

## SUPPLEMENTARY FIGURES


