# Correction: Cbl-b inhibits P-gp transporter function by preventing its translocation into caveolae in multiple drug-resistant gastric and breast cancers

**DOI:** 10.18632/oncotarget.27885

**Published:** 2021-08-03

**Authors:** Ye Zhang, Xiujuan Qu, Yuee Teng, Zhi Li, Ling Xu, Jing Liu, Yanju Ma, Yibo Fan, Ce Li, Shizhou Liu, Zhenning Wang, Xuejun Hu, Jingdong Zhang, Yunpeng Liu

**Affiliations:** ^1^ Department of Medical Oncology, the First Hospital of China Medical University, Shenyang 110001, China; ^2^ Department of Surgical Oncology and General Surgery, the First Hospital of China Medical University, Shenyang 110001, China; ^3^ Department of Medical Respiratory, the First Hospital of China Medical University, Shenyang 110001, China

**This article has been corrected:** Due to errors during figure assembly, the fluorescence images in Figure 3E are accidental duplicates of those in Figures 1D and 2D. The corrected Figure 3, as well as an updated Figure 2 showing a correctly paired “control” and “+ DOX”, are shown below. In addition, the title of Table 1 should be “breast cancer”, not “gastric cancer.” All revisions presented were obtained with the original data. The authors declare that these corrections do not change the results or conclusions of this paper.

Original article: Oncotarget. 2015; 6:6737–6748. 6737-6748. https://doi.org/10.18632/oncotarget.3253

**Figure 2:**
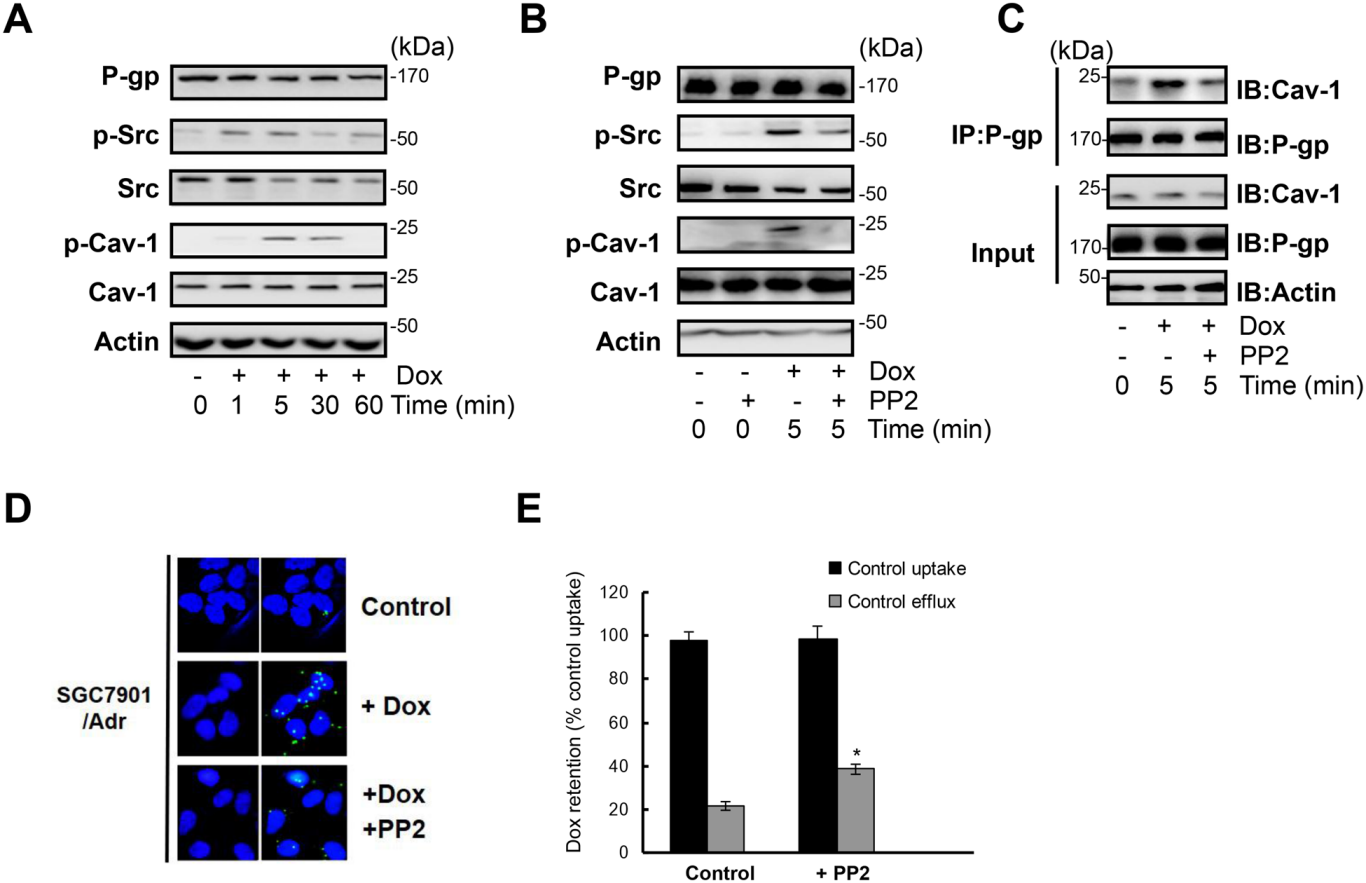
c-Src dependent Cav-1 phosphorylation promoted the translocation of P-gp into caveolae. (**A**) SGC7901/Adr cells were treated with or without 20 μg/ml Dox for 1, 5, 30, 60 min and the expression of P-gp, p-Src, Src, p-Cav-1, Cav-1 and Actin was detected by western blotting. (**B**) Cells were incubated with the Src inhibitor PP2 (10 μmol/l) for 2 h, treated with 20 μg/ml Dox for 5 min, and the expression of P-gp, p-Src, Src, p-Cav-1, Cav-1 and Actin was detected by western blotting. (**C**) SGC7901/Adr cells were pretreated with 10 μmol/l PP2 for 2 h followed by Dox treatment, and P-gp was immunoprecipitated and Cav-1 was analyzed western blotting. (**D**) *In situ* PLA in SGC7901/Adr cells pretreated with or without 10 μmol/l PP2 for 2 h, and then incubated with 20 μg/ml Dox for 5 min. Primary mouse and rabbit antibodies against P-gp and Cav-1 were combined with secondary PLA probes. (**E**) SGC7901/Adr cells were pretreated with 10 μmol/l PP2 for 2 h, followed by 20 μg/ml Dox and assessment of R-123 uptake and efflux.

**Figure 3:**
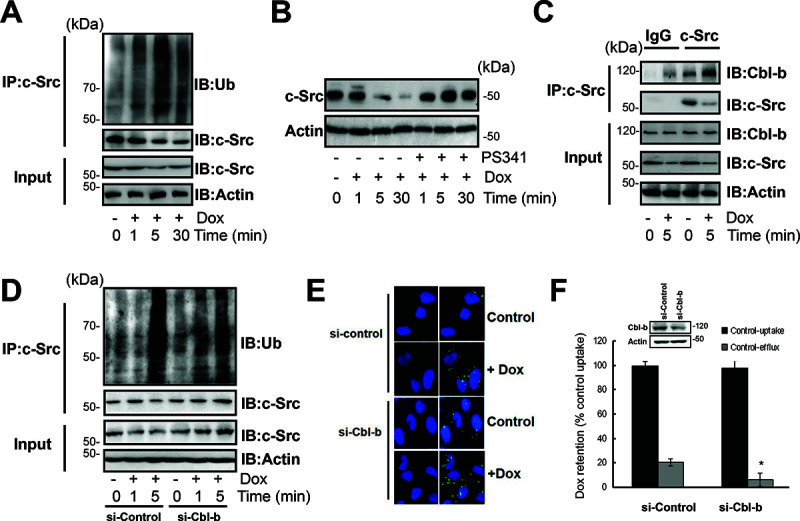
Cbl-b inhibited the translocation of P-gp into caveolae by inducing the ubiquitination and degradation of c-Src. (**A**) SGC7901/Adr cells were exposed to 20 μg/ml Dox for 1, 5, 30 min, c-Src was immunoprecipitated and ubiquitin was analyzed by western blotting. (**B**) SGC7901/Adr cells were incubated with PS341 (5 nmol/l) for 12 h, then treated with 20 μg/ml Dox for 5 min, and the expression of the Src protein was analyzed by western blotting. (**C**) SGC7901/Adr cells were treated with or without 20 μg/ml Dox for 5, c-Src was immunoprecipitated and Cbl-b was analyzed by western blotting. (**D**) SGC7901/Adr cells were transiently transfected with Cbl-b siRNA (si-Cbl-b) for 48 h, followed by 20 μg/ml Dox for 1 or 5 min, c-Src was immunoprecipitated and ubiquitin was analyzed by western blotting. (**E**) SGC7901/Adr cells were transiently transfected with Cbl-b siRNA (si-Cbl-b) for 48 h and analyzed by PLA after incubation with 20 μg/ml Dox for 5 min. Primary mouse and rabbit antibodies against the P-gp and Cav-1 were combined with secondary PLA probes. (**F**) SGC7901/Adr cells were transiently transfected with Cbl-b siRNA (si-Cbl-b) for 48 h, followed by 20 μg/ml Dox and analysis of Dox uptake and efflux.

**Table 1:** Relationship between the expression of Cbl-b and clinico-pathological characteristics of P-gp-positive breast cancer patients

**Clinico-pathological characteristics**		**Number**	**Cbl-b expression**		***p* value **
			**Negative**	**Positive**	
Age (years)					
	≤ 35	7	5	2	
	> 35	114	43	71	0.112
Tumour size (cm)					
	≤ 2	19	9	10	
	> 2	102	39	63	0.455
pN stage					
	0	58	19	39	
	1–3	63	29	34	0.136
Histology grade					
	1 + 2	87	33	54	
	3	34	15	19	0.532
ER/PR status^§^					
	Negative	59	19	40	
	Positive	62	29	33	0.101
HER2 status^¶^					
	Negative	39	22	17	
	Positive	82	26	56	**0.009^1^**

^1^Two-sided *p* value; values shown in bold are statistically significant.

^§^ER/PR status: oestrogen receptor or progesterone receptor status by immunohistochemistry (IHC); negative: ER and PR double negative; positive: ER or PR positive.

^¶^HER2 status: HER2-positive status is IHC 3+ or fluorescence *in situ* hybridisation (FISH) positive; HER2-negative status is IHC 0, 1+ or FISH negative.

HER2, Human epidermal growth factor receptor 2.

