# Supplementary material for: Cbl-b inhibits P-gp transporter function by preventing its translocation into caveolae in multiple drug-resistant gastric and breast cancers
Source: Oncotarget. 2015 Feb 17;6(9):6737–48. doi: 10.18632/oncotarget.3253 (PMC4466646; doi:10.18632/oncotarget.3253)
Supplement: Supplementary file 1 [file oncotarget-06-6737-s001.pdf]

SUPPLEMENTARY FIGURES

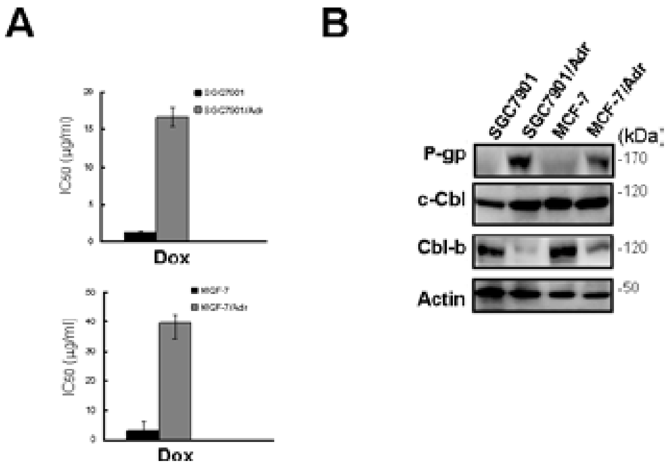

Supplementary Figure 1: Drug resistance and P-gp, c-Cbl, and Cbl-b levels in MDR cell lines. (A) IC_{50} values for Dox in SGC7901, SGC7901/Adr, MCF-7, and MCF-7/Adr cells. IC_{50} values were calculated as described in the Materials and methods section. **(B)** Levels of P-gp, c-Cbl, Cbl-b, and actin were examined by western blotting. Cells were maintained in Dox (1 µg/ml); 7 days later, cells were released to drug-free medium. Actin was used as the internal control.

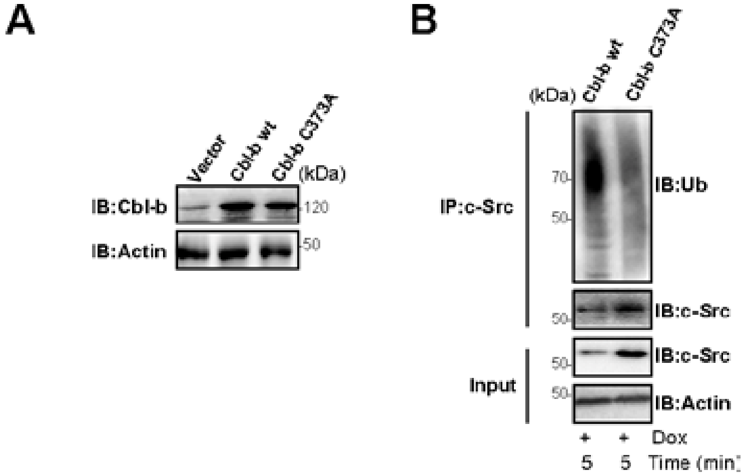

Supplementary Figure 2: Ubiquitination of c-Src requires Cbl-b. (A) Cbl-b expression was analyzed by western blotting in SGC7901/Adr cells stably transfected with a Cbl-b wild type plasmid or the Cbl-b C373A mutant. **(B)** SGC7901/Adr cells were stably transfected with a Cbl-b wild type plasmid or Cbl-b C373A, followed by 20 µg/ml Dox treatment for 5 min. c-Src was immunoprecipitated and its ubiquitination was detected by western blotting.

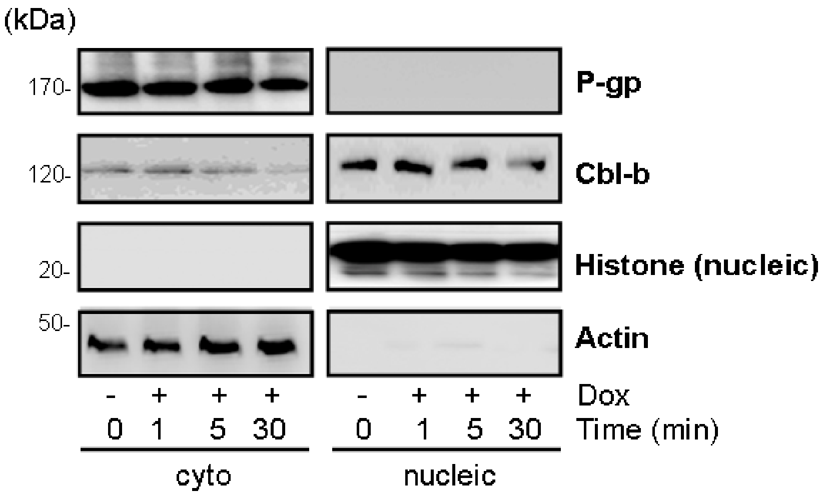

Supplementary Figure 3: Expression levels of Cbl-b and P-gp in SGC7901/Adr cells. Western blot analysis of cytoplasmic and nuclear fractions from SGC7901/Adr cells, which were treated with 2 µg/ml Dox for 1, 5, and 30 min. Nuclear and cytoplasmic fractionation was detected by total histone and actin expression, respectively.

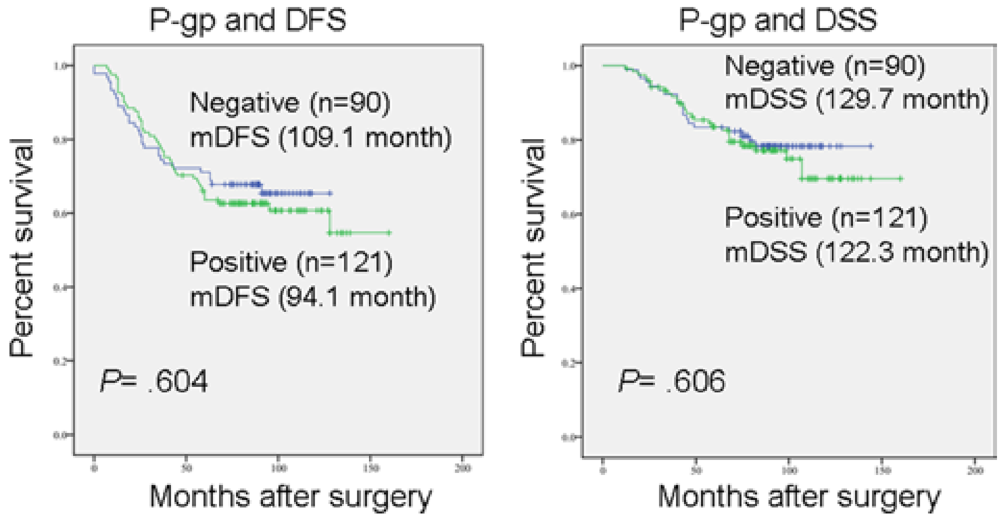

Supplementary Figure 4: Disease-specific survival rates of breast cancer patients with positive or negative for P-gp were estimated with the Kaplan–Meier method and log-rank test.
